# Bis{2-[(2-cyano­phen­yl)imino­meth­yl]phenolato}copper(II)

**DOI:** 10.1107/S160053680803417X

**Published:** 2008-11-13

**Authors:** Rong Xia, Hai-Jun Xu, Han Wang

**Affiliations:** a Ordered Matter Science Research Center, College of Chemistry and Chemical Engineering, Southeast University, Nanjing 210096, People’s Republic of China

## Abstract

In the title mononuclear copper(II) complex, [Cu(C_14_H_9_N_2_O)_2_], the Cu^II^ atom, situated on an inversion centre, shows a slightly distorted square-planar geometry and is coordinated by the N and O atoms from two deprotonated symmetry-related Schiff base ligands. The Cu—N and Cu—O bond lengths are 2.009 (2) and 1.888 (2) Å, respectively. The dihedral angle between the cyano­phenyl rings and phenolate rings is 42.28 (13)°.

## Related literature

For Schiff base complexes with copper(II) and nickel(II), see: Gong *et al.* (2008 ▶); Kitaura *et al.* (2004 ▶); Marganian *et al.* (1995 ▶). For bond-length data, see: Jian *et al.* (2004 ▶); Ünver (2002 ▶). For a related structure, see: Xia *et al.* (2008 ▶).
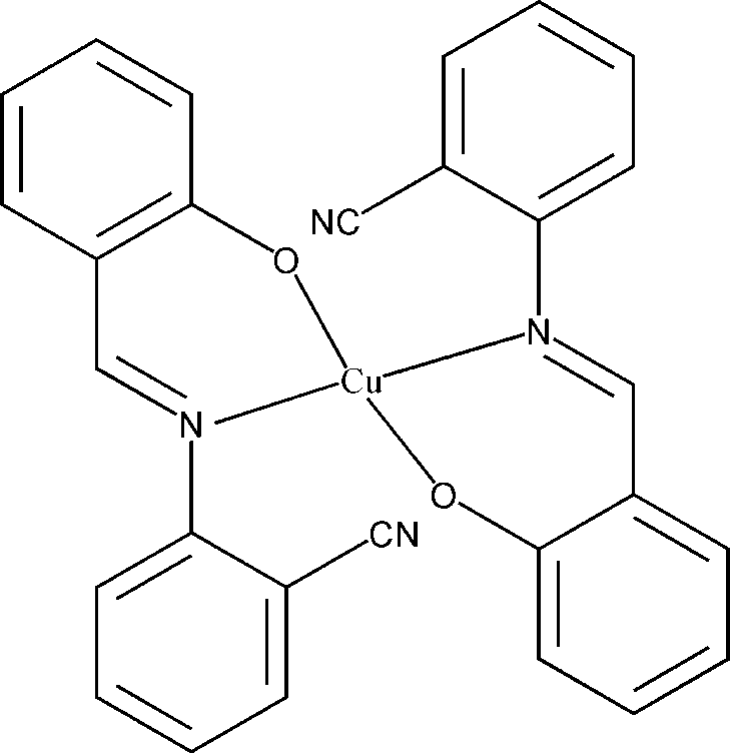


## Experimental

### 

#### Crystal data

[Cu(C_14_H_9_N_2_O)_2_]
*M* *_r_* = 506.00
Monoclinic, 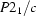

*a* = 9.698 (3) Å
*b* = 11.403 (3) Å
*c* = 10.889 (3) Å
β = 107.570 (11)°
*V* = 1148.0 (6) Å^3^
*Z* = 2
Mo *K*α radiation
μ = 0.99 mm^−1^
*T* = 293 (2) K
0.15 × 0.10 × 0.10 mm

#### Data collection

Rigaku Mercury2 diffractometer
Absorption correction: multi-scan (*CrystalClear*; Rigaku, 2005 ▶) *T*_min_ = 0.909, *T*_max_ = 1.000 (expected range = 0.824–0.906)
11307 measured reflections
2629 independent reflections
1952 reflections with *I* > 2σ(*I*)
*R*_int_ = 0.057

#### Refinement

*R*[*F* ^2^ > 2σ(*F* ^2^)] = 0.050
*wR*(*F* ^2^) = 0.125
*S* = 1.09
2629 reflections
160 parameters
H-atom parameters constrained
Δρ_max_ = 0.26 e Å^−3^
Δρ_min_ = −0.43 e Å^−3^

### 

Data collection: *CrystalClear* (Rigaku, 2005 ▶); cell refinement: *CrystalClear*; data reduction: *CrystalClear*; program(s) used to solve structure: *SHELXS97* (Sheldrick, 2008 ▶); program(s) used to refine structure: *SHELXL97* (Sheldrick, 2008 ▶); molecular graphics: *SHELXTL* (Sheldrick, 2008 ▶); software used to prepare material for publication: *SHELXL97*.

## Supplementary Material

Crystal structure: contains datablocks I, global. DOI: 10.1107/S160053680803417X/gw2050sup1.cif

Structure factors: contains datablocks I. DOI: 10.1107/S160053680803417X/gw2050Isup2.hkl

Additional supplementary materials:  crystallographic information; 3D view; checkCIF report

## Figures and Tables

**Fig. 1.**
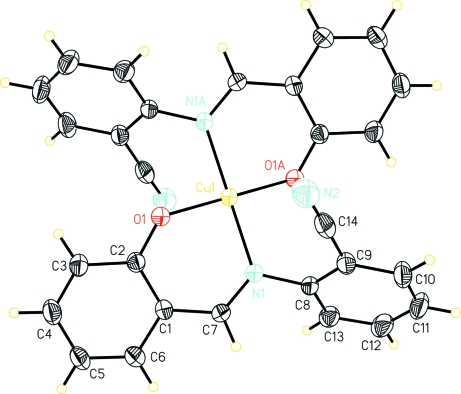
Molecular view of the title compound with the atom-labelling scheme. Displacement ellipsoids are drawn at the 30% probability level. H atoms are represented as small spheres of arbitrary radii. [Symmetry code: (i) 2 - x, 1 - y, -z]

## supplementary crystallographic information

### Comment

Metal derivatives of Schiff bases have been studied extensively, and copper(II) and nickel(II) complexes play an important role in both synthetic and structural research. These complexes have received much attention in recent years (Marganian *et al.*, 1995; Kitaura *et al.* 2004). We have reported previously the crystal structures of monomeric Schiff base complexes of Ni^II^ (Gong *et al.*, 2008). As an a continuation of our research on the synthesis and structure of transition metal complexesof Schiff base compounds, we here report the results of the reaction of copper(II) with the didentate ligand 2-(2-cyanophenyliminomethyl)phenol, forming the title compound (I).

Fig.1 shows the molecular structure of the title compound. The copper(II) is coordinated by the two imine N and two phenolate O atoms of the two Schiff base ligands in a slightly distorted square-planar geometry in a *trans* arrangement. 2-(2-cyanophenyliminomethyl)phenol loses a proton form the hydroxyl group and acts as a singlely charged bidentate ligand coordinating to Copper(II) through the phenolate O and imine N atoms. The dihedral angle between the C1—C6 and C8—C13 benzene rings is 42.28 (0.13)°. The N1—Cu1—O1 bond angles is 90.78 (9)°. The two equivalent Cu–N and Cu–O distances are 2.009 (2)Å and 1.888 (2) Å, respectively. All these parameters conform to values in other square-planar-coordinated copper(II) compounds(Jian *et al.*, 2004; Ünver 2002).

### Experimental

2-(2-Cyanophenyliminomethyl)phenol was prepared according to the literature(Xia *et al.*, 2008).CuCl_2_.2H_2_O(17. mg, 0.1 mmol) in methanol (5 ml) was added to the solution of 2-(2-cyanophenyliminomethyl)phenol (22.2 mg, 0.1 mmol)in the methanol (5 ml), pH of themixture was adjusted to 8–9 and stirred for 4 h. The filtrate was kept at room temperature for about two weeks, and green block crystals of (I) for X-ray single-crystal investigation were obtained.

### Refinement

All H atoms attached to C atoms were fixed geometrically and treated as riding with C—H = 0.93 Å and *U*_iso_(H) = 1.2*U*_eq_(C).

### Crystal data

**Table:**

[Cu(C_14_H_9_N_2_O)_2_]	*F*_000_ = 518
*M**_r_* = 506.00	*D*_x_ = 1.464 Mg m^−^^3^
Monoclinic, *P*2_1_/*c*	Mo *K*α radiation λ = 0.71073 Å
Hall symbol: -P 2ybc	Cell parameters from 2373 reflections
*a* = 9.698 (3) Å	θ = 3.1–27.4º
*b* = 11.403 (3) Å	µ = 0.99 mm^−^^1^
*c* = 10.889 (3) Å	*T* = 293 (2) K
β = 107.570 (11)º	Block, red
*V* = 1148.0 (6) Å^3^	0.15 × 0.10 × 0.10 mm
*Z* = 2	

### Data collection

**Table:**

Rigaku Mercury2 diffractometer	2629 independent reflections
Radiation source: fine-focus sealed tube	1952 reflections with *I* > 2σ(*I*)
Monochromator: graphite	*R*_int_ = 0.057
Detector resolution: 13.6612 pixels mm^-1^	θ_max_ = 27.5º
*T* = 293(2) K	θ_min_ = 3.1º
ω scans	*h* = −12→12
Absorption correction: multi-scan(CrystalClear; Rigaku, 2005)	*k* = −14→14
*T*_min_ = 0.909, *T*_max_ = 1.000	*l* = −14→14
11307 measured reflections	

### Refinement

**Table:**

Refinement on *F*^2^	Secondary atom site location: difference Fourier map
Least-squares matrix: full	Hydrogen site location: inferred from neighbouring sites
*R*[*F*^2^ > 2σ(*F*^2^)] = 0.050	H-atom parameters constrained
*wR*(*F*^2^) = 0.125	*w* = 1/[σ^2^(*F*_o_^2^) + (0.0516*P*)^2^ + 0.2943*P*] where *P* = (*F*_o_^2^ + 2*F*_c_^2^)/3
*S* = 1.09	(Δ/σ)_max_ < 0.001
2629 reflections	Δρ_max_ = 0.26 e Å^−^^3^
160 parameters	Δρ_min_ = −0.43 e Å^−^^3^
Primary atom site location: structure-invariant direct methods	Extinction correction: none

### Special details

**Table:**

Geometry. All e.s.d.'s (except the e.s.d. in the dihedral angle between two l.s. planes) are estimated using the full covariance matrix. The cell e.s.d.'s are taken into account individually in the estimation of e.s.d.'s in distances, angles and torsion angles; correlations between e.s.d.'s in cell parameters are only used when they are defined by crystal symmetry. An approximate (isotropic) treatment of cell e.s.d.'s is used for estimating e.s.d.'s involving l.s. planes.
Refinement. Refinement of *F*^2^ against ALL reflections. The weighted *R*-factor *wR* and goodness of fit *S* are based on *F*^2^, conventional *R*-factors *R* are based on *F*, with *F* set to zero for negative *F*^2^. The threshold expression of *F*^2^ > σ(*F*^2^) is used only for calculating *R*-factors(gt) *etc*. and is not relevant to the choice of reflections for refinement. *R*-factors based on *F*^2^ are statistically about twice as large as those based on *F*, and *R*- factors based on ALL data will be even larger.

### Fractional atomic coordinates and isotropic or equivalent isotropic displacement parameters (Å^2^)

**Table:**

	*x*	*y*	*z*	*U*_iso_*/*U*_eq_	
Cu1	1.0000	0.5000	0.0000	0.03878 (18)	
N1	0.9612 (2)	0.6674 (2)	0.0368 (2)	0.0383 (5)	
O1	1.0810 (2)	0.46983 (18)	0.1778 (2)	0.0469 (5)	
C8	0.8569 (3)	0.7359 (3)	−0.0563 (3)	0.0400 (6)	
C1	1.1420 (3)	0.6686 (3)	0.2473 (3)	0.0416 (7)	
C7	1.0377 (3)	0.7214 (3)	0.1397 (3)	0.0416 (7)	
H7A	1.0234	0.8018	0.1439	0.050*	
C9	0.7154 (3)	0.6925 (3)	−0.1043 (3)	0.0469 (7)	
C2	1.1568 (3)	0.5450 (3)	0.2631 (3)	0.0403 (7)	
C4	1.3310 (4)	0.5785 (3)	0.4717 (3)	0.0587 (9)	
H4A	1.3938	0.5479	0.5471	0.070*	
C6	1.2221 (3)	0.7432 (3)	0.3469 (3)	0.0535 (8)	
H6A	1.2098	0.8239	0.3373	0.064*	
C13	0.8922 (4)	0.8413 (3)	−0.1019 (3)	0.0507 (8)	
H13A	0.9852	0.8715	−0.0698	0.061*	
C14	0.6811 (3)	0.5801 (3)	−0.0602 (3)	0.0539 (8)	
C10	0.6108 (4)	0.7564 (4)	−0.1967 (3)	0.0624 (10)	
H10A	0.5162	0.7291	−0.2270	0.075*	
C12	0.7880 (5)	0.9012 (3)	−0.1957 (4)	0.0670 (10)	
H12A	0.8126	0.9711	−0.2280	0.080*	
C5	1.3176 (4)	0.6996 (3)	0.4574 (3)	0.0601 (9)	
H5A	1.3720	0.7496	0.5211	0.072*	
C3	1.2547 (4)	0.5035 (3)	0.3782 (3)	0.0507 (8)	
H3A	1.2678	0.4231	0.3909	0.061*	
N2	0.6591 (4)	0.4905 (3)	−0.0242 (4)	0.0716 (9)	
C11	0.6491 (5)	0.8599 (4)	−0.2422 (4)	0.0729 (12)	
H11A	0.5805	0.9021	−0.3049	0.087*	

### Atomic displacement parameters (Å^2^)

**Table:**

	*U*^11^	*U*^22^	*U*^33^	*U*^12^	*U*^13^	*U*^23^
Cu1	0.0424 (3)	0.0348 (3)	0.0347 (3)	0.0037 (2)	0.0050 (2)	−0.0003 (2)
N1	0.0399 (13)	0.0373 (13)	0.0349 (12)	0.0036 (10)	0.0069 (10)	0.0010 (10)
O1	0.0581 (14)	0.0381 (12)	0.0378 (12)	0.0047 (9)	0.0046 (10)	−0.0004 (8)
C8	0.0428 (16)	0.0400 (16)	0.0340 (15)	0.0077 (12)	0.0066 (12)	−0.0006 (12)
C1	0.0385 (16)	0.0445 (17)	0.0373 (16)	0.0018 (12)	0.0044 (12)	−0.0004 (12)
C7	0.0445 (17)	0.0332 (15)	0.0433 (16)	0.0020 (12)	0.0074 (13)	−0.0001 (12)
C9	0.0429 (18)	0.0526 (19)	0.0407 (17)	0.0068 (14)	0.0059 (13)	−0.0004 (14)
C2	0.0407 (17)	0.0443 (16)	0.0349 (15)	0.0040 (13)	0.0098 (13)	0.0009 (12)
C4	0.050 (2)	0.073 (3)	0.0432 (19)	0.0075 (17)	−0.0005 (15)	0.0027 (16)
C6	0.058 (2)	0.0493 (19)	0.0458 (19)	−0.0028 (15)	0.0038 (15)	−0.0048 (14)
C13	0.057 (2)	0.0476 (18)	0.0479 (19)	0.0047 (15)	0.0162 (15)	0.0054 (14)
C14	0.0386 (18)	0.071 (2)	0.0456 (19)	−0.0032 (16)	0.0030 (14)	−0.0054 (17)
C10	0.046 (2)	0.081 (3)	0.050 (2)	0.0174 (18)	−0.0011 (16)	0.0000 (18)
C12	0.092 (3)	0.051 (2)	0.055 (2)	0.0172 (19)	0.018 (2)	0.0155 (16)
C5	0.061 (2)	0.065 (2)	0.0416 (19)	−0.0074 (17)	−0.0045 (16)	−0.0060 (16)
C3	0.056 (2)	0.0516 (19)	0.0402 (17)	0.0096 (15)	0.0080 (14)	0.0061 (14)
N2	0.064 (2)	0.067 (2)	0.077 (2)	−0.0158 (16)	0.0115 (17)	0.0050 (17)
C11	0.079 (3)	0.073 (3)	0.055 (2)	0.036 (2)	0.003 (2)	0.0149 (19)

### Geometric parameters (Å, °)

**Table:**

Cu1—O1^i^	1.888 (2)	C4—C3	1.364 (4)
Cu1—O1	1.888 (2)	C4—C5	1.391 (5)
Cu1—N1^i^	2.009 (2)	C4—H4A	0.9300
Cu1—N1	2.009 (2)	C6—C5	1.371 (4)
N1—C7	1.298 (3)	C6—H6A	0.9300
N1—C8	1.429 (3)	C13—C12	1.381 (4)
O1—C2	1.313 (4)	C13—H13A	0.9300
C8—C13	1.382 (4)	C14—N2	1.137 (4)
C8—C9	1.403 (4)	C10—C11	1.374 (5)
C1—C2	1.421 (4)	C10—H10A	0.9300
C1—C6	1.413 (4)	C12—C11	1.372 (5)
C1—C7	1.429 (4)	C12—H12A	0.9300
C7—H7A	0.9300	C5—H5A	0.9300
C9—C10	1.398 (4)	C3—H3A	0.9300
C9—C14	1.443 (5)	C11—H11A	0.9300
C2—C3	1.407 (4)		
			
O1^i^—Cu1—O1	180.00 (12)	C3—C4—C5	121.9 (3)
O1^i^—Cu1—N1^i^	90.78 (9)	C3—C4—H4A	119.1
O1—Cu1—N1^i^	89.22 (9)	C5—C4—H4A	119.1
O1^i^—Cu1—N1	89.22 (9)	C5—C6—C1	121.7 (3)
O1—Cu1—N1	90.78 (9)	C5—C6—H6A	119.2
N1^i^—Cu1—N1	180.00 (13)	C1—C6—H6A	119.2
C7—N1—C8	116.8 (2)	C8—C13—C12	119.4 (3)
C7—N1—Cu1	122.03 (19)	C8—C13—H13A	120.3
C8—N1—Cu1	120.84 (18)	C12—C13—H13A	120.3
C2—O1—Cu1	125.24 (19)	N2—C14—C9	177.6 (4)
C13—C8—C9	119.5 (3)	C11—C10—C9	119.4 (3)
C13—C8—N1	122.1 (3)	C11—C10—H10A	120.3
C9—C8—N1	118.4 (3)	C9—C10—H10A	120.3
C2—C1—C6	119.5 (3)	C11—C12—C13	121.3 (4)
C2—C1—C7	122.5 (3)	C11—C12—H12A	119.3
C6—C1—C7	117.7 (3)	C13—C12—H12A	119.3
N1—C7—C1	125.9 (3)	C6—C5—C4	118.3 (3)
N1—C7—H7A	117.1	C6—C5—H5A	120.9
C1—C7—H7A	117.1	C4—C5—H5A	120.9
C8—C9—C10	120.0 (3)	C4—C3—C2	121.5 (3)
C8—C9—C14	119.1 (3)	C4—C3—H3A	119.3
C10—C9—C14	120.8 (3)	C2—C3—H3A	119.3
O1—C2—C3	119.6 (3)	C12—C11—C10	120.3 (3)
O1—C2—C1	123.2 (3)	C12—C11—H11A	119.9
C3—C2—C1	117.2 (3)	C10—C11—H11A	119.9

Symmetry codes: (i) −*x*+2, −*y*+1, −*z*.
